# Association of Major Histocompatibility Complex Polymorphism With Acute Phase Response in Broiler Chicken

**DOI:** 10.1002/vms3.70062

**Published:** 2024-10-29

**Authors:** Afra Vatankhah, Gholamreza Nikbakht Brujeni, Atefeh Esmailnejad

**Affiliations:** ^1^ Department of Microbiology and Immunology Faculty of Veterinary Medicine University of Tehran Tehran Iran; ^2^ Department of Pathobiology Faculty of Veterinary Medicine University of Shiraz Shiraz Iran

**Keywords:** acute phase proteins | chicken | inflammation | LEI0258 | major histocompatibility complex (MHC)

## Abstract

**Background:**

Stress associated with changes in host immunity occurs in response to altered environmental conditions, endogenous imbalances, infectious agents and harmful stimuli. The importance of genetic diversity in chickens has increased due to individual immune differences towards resistance and susceptibility to various stimuli.

**Objectives:**

This study aimed to investigate the association of major histocompatibility complex (MHC) polymorphism with acute phase response (APR) in Ross 308 broiler chickens.

**Methods:**

The allelic diversity of the LEI0258 microsatellite marker was determined in 120 Ross broilers. In addition, acute phase proteins (APPs), including serum amyloid A (SAA) and alpha‐1‐acid glycoprotein (AGP), were analysed as markers of the APR. Furthermore, leukocyte count and the heterophil/lymphocyte ratio (H/L ratio) were examined. The antibody response to the Newcastle disease vaccine (NDV) was also measured to assess humoral mediated immunity. Lastly, the correlation between immune responses and MHC alleles was investigated to identify the most effective alleles in a stress‐related situation.

**Results:**

A total of six alleles, ranging from 195 to 448 bp, were identified. Association study revealed a significant influence of MHC alleles on APPs in Ross population (*p* < 0.05). Notably, Allele 448 had a significant correlation with SAA concentration and the H/L ratio. Allele 207 displayed a positive association with AGP concentration, whereas Allele 195 showed a negative association. Furthermore, a significant association was observed between Allele 448 and basopenia, as well as between Allele 195 and monocytosis.

**Conclusions:**

Results confirmed the significance of MHC as a candidate gene marker for immune responses, which supports its use for vaccine design, genetic improvement of disease‐resistant traits and resource conservation in commercial broiler chickens.

## Graphical abstract

- This study revealed a significant association of chicken MHC haplotypes with the level of acute phase proteins (SAA and AGP) in response to vaccination.
- A significant correlation was observed between MHC haplotypes and the H/L ratio, as well as basopenia and monocytosis.
- Important role of chicken MHC as a candidate gene marker for adaptive immune responses.

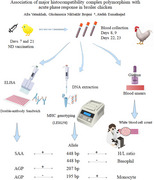

## 1 Introduction

Pathological stimuli are recognized by the innate immune system to elicit both immediate defence and long‐lasting adaptive immunity. It has been reported that different parts of the innate immune response, including inflammation, have the potential to impact disease resistance or susceptibility, consequently influencing disease outcomes within a population (Collisson, Griggs, and Drechsler 2017; Silva and Gallardo 2020; Smith et al. 2015). A major part of innate immunity is mediated by the acute phase response (APR), which is triggered by infection, stress, trauma or inflammation (Cray, Zaias, and Altman 2009; Eckersall et al. 2010; Gruys et al. 2005). APR can be identified and monitored by several laboratory tests, such as measuring the concentration of plasma proteins known as acute phase proteins (APPs). These proteins are largely synthesized by the liver and classified as either positive or negative based on their increased or decreased synthesis, respectively. The plasma concentration of APPs is proportional to the severity and/or extent of tissue damage and inflammatory states (Chamanza et al. 1999; Gruys, Obwolo, and Toussaint 1994).

The extensive biological function of APPs in chickens has been reviewed (O'Reilly and Eckersall 2014). Serum amyloid A (SAA) and alpha‐1‐acid glycoprotein (AGP) are major and moderately positive APPs that play crucial immunomodulatory roles in the initial stages of inflammation and infection in chickens (Chamanza et al. 1999; O'Reilly and Eckersall 2014). The measurement of APPs as well as the heterophil/lymphocyte (H/L) ratio can be used for the monitoring of various aspects of health and as indicators for the assessment of stress in chickens (Cray, Zaias, and Altman 2009; Janmohammadi et al. 2020; Ohara et al. 2015; Shini et al. 2008).

Chickens show individual differences in immune responses to various stimuli, which could be related to genetic diversity in immune response genes. The best characterized genetic control of disease resistance and immune response in the chicken is that associated with the major histocompatibility complex (MHC), which plays a crucial role in how their immune systems recognize and respond to pathogens (Lamont 1998; Zekarias et al. 2002). The chicken MHC‐B region is located on Microchromosome 16 and consists of a linkage group of three polymorphic regions: BF (class I), BL (class II), and BG (Miller et al. 2016). Although most genes in the MHC‐B are primarily linked to adaptive immunity, it is worth mentioning that there are also genes that play a significant role in innate immunity or in the combination of innate and adaptive immunity, commonly known as the adaptate (Kaufman 2022), such as the gene encoding a leukotriene B4 receptor for potent chemoattractants involved in inflammation (Saeki and Yokomizo 2017), the gene involved with the complement cascade and the genes that are involved with natural‐killer (NK) or γδ T cells. The TRIM region (a region with tripartite motif) is located between BF–BL and the BG region. Both the TRIM and BG regions have multigene families that are almost certainly involved in innate and adaptate responses (Fulton et al. 2016; Kaufman 2022). It has been shown that the BG1 locus has a highly significant influence on the occurrence of Marek's disease (Goto, Wang, and Taylor 2009), which provided evidence that genes other than BF/BL also contribute to MHC‐B‐linked disease resistance. Therefore, MHC has an extensive regulatory effect on many immune response traits, such as MHC genes’ impact on antibody responses (Dunnington et al. 1996; Kean et al. 1994; Loudovaris, Brandon, and Fahey 1990; Zhou and Lamont 2003), cytokine production (Gehad et al. 1999; Silva and Gallardo 2020), activity of cytotoxic and NK cells (Schat, Taylor, and Briles 1994) and acceleration of leukocyte infiltration into infection sites (Saeki and Yokomizo 2017).

The MHC‐B is a minimal essential gene set that differs from the mammalian MHC. Despite its simplicity, it retains the essential counterpart genes of the mammalian MHC, allowing for a strong association to be detected between the MHC‐B and resistance or susceptibility to disease (Kaufman 2000). This association, along with variability in response to vaccines and production and reproduction traits, has been linked to MHC‐B haplotypes identified using allelic variants of the LEI0258 microsatellite marker in different chicken populations (Esmailnejad, Brujeni, and Badavam 2017; Haunshi et al. 2020; Lwelamira et al. 2008; Mpenda, Tiambo, and Kyallo 2020; Nassar 2021; Schou, Labouriau, and Permin 2010). LEI0258 microsatellite, a highly polymorphic tandem repeat genetic marker, is located between the BF and BG regions and its usefulness in reflecting the variability of the MHC‐B region in immune genotyping studies has been demonstrated (Chang et al. 2012; Fulton et al. 2006; Guangxin et al. 2014; Izadi et al. 2011; Lima‐Rosa et al. 2005).

In this study, we hypothesized that genetic variation in the MHC‐B region may influence APP levels resulting from immunization‐induced inflammation. Our aim was to investigate the possible association between MHC alleles (LEI0258) and APP levels (SAA and AGP), as well as leukocytes, which serve as indicators of APR and inflammation, in Ross broiler chickens following Newcastle disease (ND) vaccination.

## 2 Materials and Methods

### 2.1 Experimental Design and Sampling

A total of 120 one‐day‐old Ross 308 broiler chicks were obtained from a commercial breeder flock. All birds were fed with commercial feed based on Ross 308 farming standard protocol. The light, temperature and ventilation were controlled and adjusted according to management guide recommendations. On Days 7 and 21, all chickens were injected intramuscularly with the inactivated oil emulsion of ND vaccine (IND) (CEVAC Broiler ND K, Hungary, Cat. 140061). The vaccine contained inactivated La Sota strain of ND virus, oil adjuvant and merthiolate preservative. Blood samples (1–1.5 mL per bird) were collected from all chickens at six different sampling times. For investigating the kinetics of the APP response, 20 samples were randomly collected before and after first and second vaccinations (24 and 48 h). For estimating the antibody response to vaccine, 20 samples were collected at Days 6 (1 day before first vaccination) and 28 (1 week after second vaccination). For association analysis, all chickens (120) were sampled 24 h after the first vaccination. Blood samples were drawn by venipuncture from the wing vein and stored in 2 mL K2‐EDTA anticoagulant collection tubes (Fartest Farzaneh Arman, Iran) based on the standard protocol and then centrifuged at 4000 × *g* for 10 min, to obtain plasma. Collected plasma samples were stored at −20°C until use. The study was approved by the Institutional Ethical Committee of the (anonymized according to journal request).

### 2.2 Assessment of Antibody Titres Against the Vaccine

Twenty chicks were sampled for estimating the antibody response to vaccine at Days 6 (1 day before first vaccination) and 28 (1 week after second vaccination). Antibody titre against INDs was measured using haemagglutination inhibition (HI) test. Four haemagglutination units of antigen (HA) and twofold diluted serum were used. HI titres were expressed as reciprocal log_2_ values of the highest serum dilution causing complete inhibition of haemagglutination.

### 2.3 DNA Extraction and MHC Genotyping

Genomic DNA was extracted from whole blood using AccuPrep Genomic DNA Extraction Kit (Bioneer, Korea, Cat.17151). MHC alleles were identified using LEI0258 microsatellite. The LEI0258 alleles were determined by PCR‐based fragment analysis as previously described (Esmailnejad, Brujeni, and Badavam 2017).

### 2.4 Differential White Blood Cell (WBC) Count and H/L Ratios

At each sampling time point, blood smears were made and stained with the May‐Grunwald Giemsa stain. Differential WBC counts, including granular (heterophils, eosinophils and basophils) and nongranular (lymphocytes and monocytes), were done on smears according to the Leishman staining technique (Gross and Siegel 1983; Ohara et al. 2015).

### 2.5 Assessment of APR

APR was assessed by measuring the quantitative level of APPs in the plasma samples. SAA and AGP levels were measured by the enzyme‐linked immunosorbent assay (ELISA) kits for chicken (Gallus), obtained from Cloud‐Clone Corp. (TX, USA, Cat. SEA885Ga & SEA816Ga). Procedures for measurements were conducted according to the manufacturer's instructions.

### 2.6 Statistical Analysis

The observed and effective allele numbers, allele and genotype frequencies, expected and observed heterozygosity and homozygosity for LEI0258 locus were estimated using Popgene software version 1.32.0 (Yeh et al. 1997). The amount of gene diversity in the population was measured by the number of alleles and the unbiased expected heterozygosity, according to the formula proposed by Nei (1973). Deviation from Hardy–Weinberg equilibrium (HWE) was also estimated using likelihood ratio test in this population. Association of LEI0258 alleles with immune responses was analysed using the model:

$$Y_{i}=\mu +\sum b_{j}f_{ij}+\varepsilon_{i}$$

where *Y_i_* is a dependent variable for specific trait in *i*th chicken; *μ* is a general mean; *f_ij_* is the copy number of the LEI0258 *j*th allele in the *i*th chicken; *b_j_* is half the substitution effect for the LEI0258 *j*th allele; and *ε_i_* is the residual effect for the *i*th chicken with variance. For each allele, all individuals were considered a non‐carrier (0) or carrier (1), and then, single‐band analysis was carried out to determine the coefficient effect of each allele. The most frequent allele was designated as the reference, and the association study was evaluated using multivariate regression analysis and GLM procedures (SPSS Ver. 21).

## 3 Results

### 3.1 LEI0258 Microsatellite Allelic Variability

In the studied population, six LEI0258 microsatellite alleles ranging from 195 to 448 bp were identified. Allele 385 bp had the highest (42.86%), and alleles 195 and 300 bp (7.14%) had the lowest frequencies. According to published data, the best B haplotype match for LEI0258 alleles found in this study was as follows: BW3 (195), B13 (207), B5 (300), BC (362), B13.1 (385) and B6 (448) (Fulton et al. 2006). Thirteen genotypes (2 homozygous and 11 heterozygous) were found in this population, of which genotype 207/385 was the most frequent (25.71%) and genotype 362/362 was the least (0.95%) (Table 1).

**TABLE 1:** LEI0258 genotype frequency in Ross 308 broiler chicken.

Locus	Genotypes (bp)	Frequency (%)
**LEI0258**	(207/195)	5.71
	(385/385)	14.29
	(362/362)	0.95
	(385/195)	7.62
	(385/207)	25.71
	(385/300)	1.90
	(385/362)	16.19
	(362/300)	1.905
	(362/195)	1.90
	(448/207)	7.62
	(448/300)	4.76
	(448/362)	6.67
	(448/385)	4.76
**Total**	13	100

### 3.2 Antibody Titre, Stress Index and the Level of APPs in Response to Vaccination

HI titres showed that post‐vaccinated chickens at Day 28 had a highly significant elevation in their response to IND (5.50 ± 1.05) compared to the pre‐vaccination at Day 6 (2.33 ± 0.52) (*p* ≤ 0.01).

**FIGURE 1:**
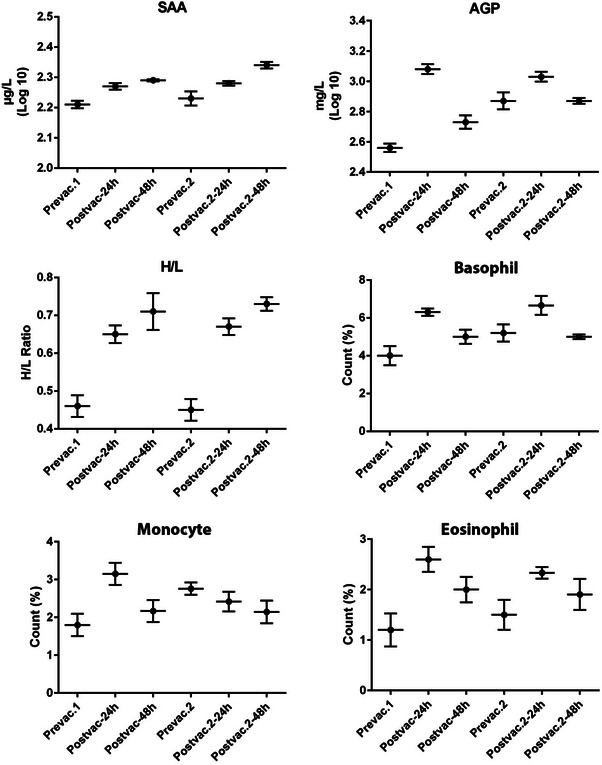
Leukocytes means in per cent, H/L ratio, SAA and AGP levels at pre‐vaccination and 1 and 2 days after first and second vaccinations. AGP, alpha‐1‐acid glycoprotein; H/L, heterophil/lymphocyte; SAA, serum amyloid A.

The blood H/L ratios and APPs (SAA and AGP) levels in plasma were assessed before and after first and second vaccinations at 24 and 48 h thereafter (Table 2). In the first and second stages of vaccinations, the H/L ratio increased 24 and 48 h after both vaccinations (*p* ≤ 0.05) (Figure 1). A significant positive association was found between SAA levels and H/L ratios at 24 and 48 h after both stages of vaccination.

**TABLE 2:** Significant association of LEI0258 alleles with fold increased acute phase proteins (APPs) levels, H/L ratio and leukocytes differential count in Ross 308 broiler chicken.

Immune traits	Fold change (Mean ± SD)	LEI0258 (bp)	Allele effect^a^	SE	*p* value
SAA	1.205 ± 0.069	448	0.385	0.170	0.026
AGP	3.390 ± 0.120	195	−1.072	0.361	0.010
		207	0.637	0.153	0.046
Basophil	1.342 ± 0.084	448	−0.894	0.207	0.042
Monocyte	1.376 ± 0.117	195	0.565	0.352	0.021
H/L	2.195 ± 0.099	448	0.636	0.244	0.026

Abbreviations: AGP, alpha‐1‐acid glycoprotein; H/L, heterophil/lymphocyte; SAA, serum amyloid A; SE, standard error.

^a^ Estimates of effects are relative to reference allele (385 bp).

The elevation pattern of AGP demonstrated a notable difference from that of SAA. Concentrations of AGP experienced a significant increase and peaked 24 h after the first and second vaccinations. More specifically, AGP log_10_ concentration levels increased from 2.56 mg/L before vaccination to 3.08 mg/L after the first vaccination and from 2.87 to 3.03 mg/L after the second vaccination (Figure 1). SAA log_10_ concentration levels were also significantly elevated and peaked on Day 2 following both first and second vaccinations, from 2.21 to 2.29 µg/L after the first vaccination and from 2.23 to 2.34 µg/L after the second vaccination (Figure 1).

### 3.3 LEI0258 Microsatellite Allele Association With Immune Traits

**FIGURE 2:**
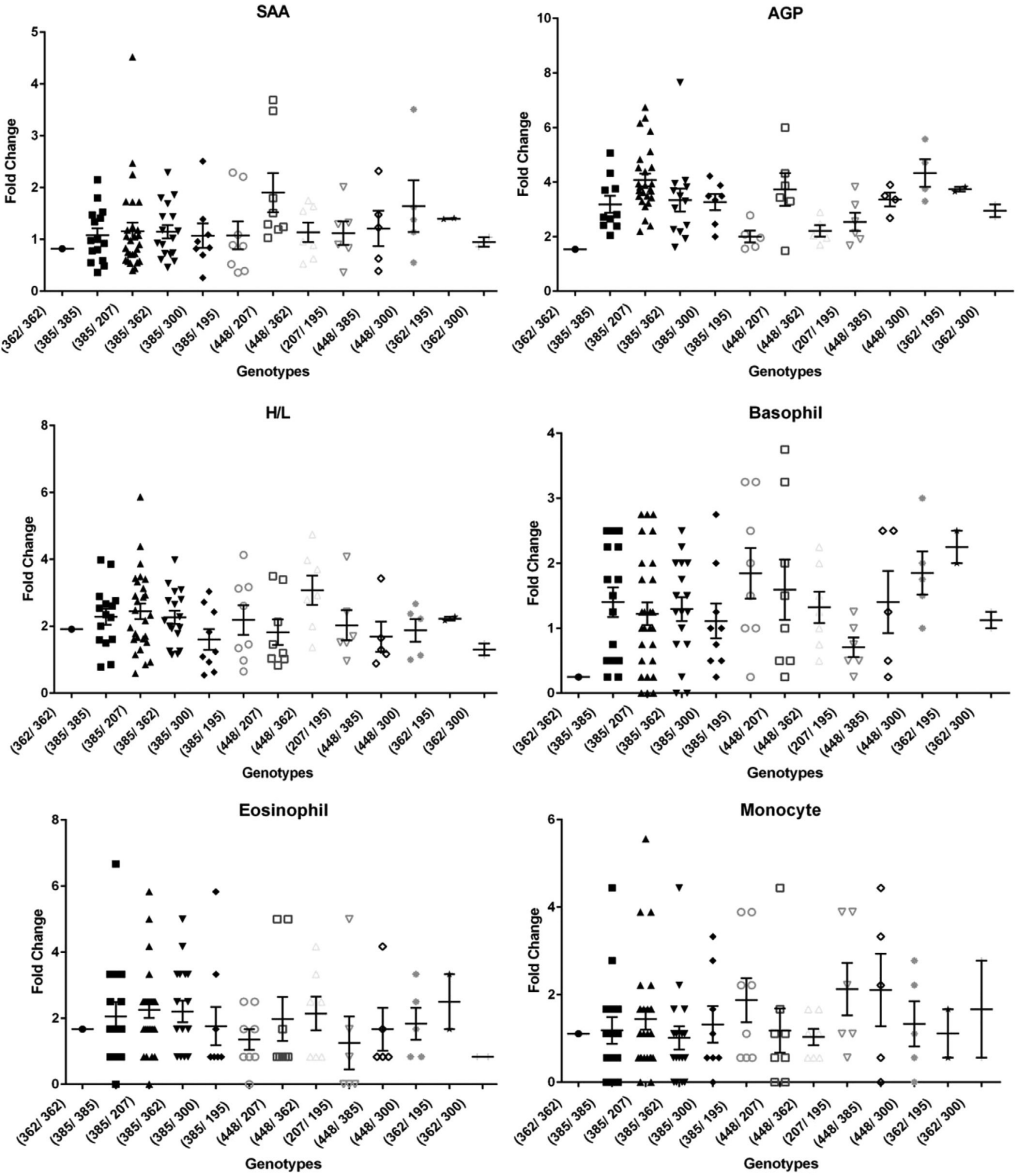
APP values and leukocyte count fold changed before and 24 h after the first vaccination for MHC genotypes (allele combinations). AGP, alpha‐1‐acid glycoprotein; APPs, acute phase proteins; H/L, heterophil/lymphocyte; MHC, major histocompatibility complex; SAA, serum amyloid A.

Average and individual APP values and leucocyte count changed for all MHC allele combinations are shown in Figure 2. Allele 385 bp was the most frequent and considered a reference allele for LEI0258 in this population. Effects of other alleles on immune traits were estimated relative to this allele (Table 2).

In total, significant association was observed between the LEI0258 microsatellite alleles with APPs (SAA and AGP) and H/L ratios. Allele 448 bp (*p *= 0.02) was positively correlated with SAA concentration against ND vaccine (NDV), in comparison to the allele 385 bp (Table 2). Alleles 195 bp (*p *= 0.01) and 207 bp (*p *= 0.04) had a negative and positive effect on AGP concentration, respectively (Table 2).

Regarding the relationship of individual microsatellite alleles with WBCs, two alleles showed significant relationships: allele 195 bp was associated with monocytosis (*p* ≤ 0.02) and allele 448 bp was correlated with basopenia (*p* ≤ 0.04). A significant correlation (*p* ≤ 0.02) between allele 448 bp and H/L ratio was also observed (Table 2).

## 4 Discussion

Stress in the poultry industry stems from mismanagement, poor nutrition, environmental factors and health conditions. It seems impossible to provide a stress‐free environment for commercial poultry flocks under intensive raising conditions. Therefore, the development of a well‐functioning immune system is essential for populations under stress in commercial poultry production system. Proper inflammatory and APRs provide broad‐spectrum protection against infections and organize long‐term adaptive immunity toward specific pathogens. In contrast, chronic and uncontrolled inflammation can be detrimental to the host and inflict severe tissue damage (Marklová 2007; Xu and Larbi 2018). During inflammation, the activation of APR is induced by signalling molecules called inflammatory cytokines and proceeds through the production of APPs in hepatocytes. These inflammatory mediators activate the fight and repair mechanisms of the innate immune response (Tizard 2013). Assessing the genetic diversity in the development of inflammation, APR and the levels of APPs in chickens has potential applications for immune analysis procedures, herd health control and vaccine effectiveness. Among the various genes involved in inflammation, MHC genes could be considered candidate genetic markers for immune responses. Therefore, exploring the relative importance of constitutive and functional diversity of MHC in inflammation could be helpful for a better understanding of the cross‐talk between the innate and adaptive branches of the immune system.

In this study, the MHC polymorphism was assessed in Ross broiler chickens using LEI0258 microsatellite marker. Several studies have used LEI0258 microsatellite to identify the MHC haplotypes in poultry (Bigabwa et al. 2022; Esmailnejad, Brujeni, and Badavam 2017; Fulton et al. 2006; Hassan et al. 2023; Lima‐Rosa et al. 2005; Manjula et al. 2021; Mpenda, Tiambo, and Kyallo 2020; Oladejo et al. 2023). To date, a total of seven LEI0258 microsatellite alleles have been identified in Ross broilers chickens (Esmailnejad, Brujeni, and Badavam 2017). With the exception of one allele (263 bp) that was not detected in our current study, the other allele types were consistent with those previously reported for the Ross population (Esmailnejad, Brujeni, and Badavam 2017). This discrepancy could indicate a decrease in genetic diversity within the Ross population, as the allele 263 bp had the lowest frequency in the previous study. These unique alleles should be considered for conservation.

In the field of disease resistance, susceptibility and vaccine effectiveness, it is important to recognize the variability of the MHC and its profound influence on immune responses. Few studies have examined the association between MHC and immune challenging tests in birds and have either failed to find an association or have found a relationship (Bonneaud et al. 2005; Bonneaud et al. 2009; Lukasch, Westerdahl, and Strandh 2017). In both Bonneaud et al. (2005) and Lukasch, Westerdahl, and Strandh (2017) studies, significant results were detected for phytohaemagglutinin (PHA) challenges, which were associated with a specific MHC genotype and with the functional MHC alleles, respectively. The association between LEI0258 microsatellite alleles and inflammation was shown by Owen et al. (2009). They discovered certain genotypes that provided resistance to mite infestation by inducing a greater degree of skin inflammation, restricting mites’ access to blood sources (Owen et al. 2009). Although the relationship between MHC and inflammation has been explored in some studies, to our knowledge, the association of MHC polymorphism with APPs as inflammatory markers has not been previously documented in broiler chickens.

The concentration of APPs in many species is linked to the severity or virulence of the pathogen, and it can be used as a marker for detecting inflammation (Eckersall. 1995; Cerón, Eckersall, and Martínez‐Subiela 2005; Chamanza et al. 1999; O'Reilly and Eckersall 2014), indicating the immediate innate immune response to stimuli such as vaccination. In the present study, IND administration resulted in an increase in SAA and AGP levels at 48 and 24 h after vaccination, respectively (Figure 1). Other studies have also shown that routine ND vaccination programs can induce an APR in both commercial layer and broiler chickens (Janmohammadi et al. 2020; Kaab, Bain, and Eckersall 2018). Kaab, Bain, and Eckersall (2018) demonstrated a significant increase in SAA levels (2.5‐fold) at 24 h and AGP levels (2‐fold) at 48 h after Newcastle and infectious bronchitis vaccination. Similarly, Janmohammadi et al. (2020) reported a 3.5‐fold increase in peak serum concentration of AGP and a 1.8‐fold increase in peak serum concentration of SAA 24 h after ND vaccination. In agreement with previous studies, in the current study SAA and AGP values increased after the first vaccination to 1.2‐ and 3.3‐fold, respectively. However, our findings indicate that 24 and 48 h after vaccination are the optimal time points for the assessment of APPs. The variation in the concentration of APPs after vaccination is due to the type of vaccine as well as individual differences in different populations.

This study was conducted to demonstrate the effects of MHC alleles on inflammation measured by APPs in broiler chickens. Our results indicated a strong relationship between some LEI0258 alleles and APP concentrations. The 448 and 207 alleles were positively associated with SAA and AGP concentrations, respectively, whereas the 195 bp allele was negatively associated with AGP concentration. The association of LEI0258 alleles with immune responses after vaccination in chickens has been investigated in previous studies (Esmailnejad, Brujeni, and Badavam 2017; Haunshi et al. 2020; Lwelamira et al. 2008; Nassar 2021). Significant association of some of the LEI0258 alleles with antibody response to NDV was reported in Tanzanian chicken ecotypes (Lwelamira et al. 2008), Indian breeds (Haunshi et al. 2020) and Egyptian local broiler breeders (Nassar 2021). Esmailnejad, Brujeni, and Badavam (2017) investigated the association of LEI0258 alleles with primary antibody responses against infectious bursal disease vaccine in broiler chickens and their results showed significant relationships, in which alleles 300, 362 and 448 bp were positively associated with higher antibody titres (Esmailnejad, Brujeni, and Badavam 2017). Our results suggest a strong association between LEI0258 alleles and immune responses to NDV and reiterate the importance of MHC in vaccine response to the NDV antigen.

The H/L ratio was measured to determine stress levels in this study. There was a remarkable increase in H/L ratio after first and second vaccinations (Figure 1). In response to nutritional and health stressors, such as low food availability, parasite infestation, migration or vaccination, the H/L ratio can be increased due to the transition of leukocytes from the marginal pool to peripheral circulation (Clark 2015). High H/L ratios may have a negative impact on some immune indices and cause higher mortality rates (Kilgas et al. 2006; Lukasch, Westerdahl, and Strandh 2017; Ochs and Dawson 2008). Our study discovered a positive correlation between SAA levels and H/L ratios. Furthermore, a highly significant positive relationship between allele 448 bp and both H/L ratios and SAA concentrations was observed. Collectively, allele 448 bp seems to be associated with elevated innate and adaptive immune responses in Ross broiler chickens.

The impact of inflammation on leukocyte circulation is intricate and may be affected by various factors, such as the underlying cause, the extent and duration of the inflammation and the individual's capability to mount an inflammatory reaction.

In this study, the count of each type of leukocyte was also analysed in relation to MHC alleles, as different types of leukocytes (heterophils, lymphocytes, monocytes, eosinophils and basophils) may be significantly affected by inflammation. The avian leukocyte response to inflammation classically results in heterophilia, lymphopenia and monocytosis (Branton et al. 1997; Jaensch and Clark 2004). There is strong published evidence to support the view that monocytosis is often seen in avian infectious or inflammatory process (Branton et al. 1997; Maxwell and Robertson 1995; Mitchell, Kettlewell, and Maxwell 1992). Basophils seem to be involved in the initial phases of acute inflammation (Buta et al. 2019); however, this does not always result in peripheral basophilia (Mitchell, Kettlewell, and Maxwell 1992). Moreover, basophil responses to inflammation in birds are variable and have not been reliably associated with specific aetiologies (Jaensch and Clark 2004). Significant basopenia has been sporadically reported in turpentine‐injected chickens (Latimer et al. 1988), and similar findings have been rarely reported in cases of chronic infections (Gross 1984). In our study, we discovered a significant association between allele 448 bp and basopenia, as well as allele 195 bp and monocytosis. This genetic linkage with the immune response may be attributed to the direct influence of the gene itself, or it may be influenced by other genes that are in linkage disequilibrium with the first gene and the interactions that occur between different components of the immune pathways.

## 5 Conclusion

Immune responses are complex traits influenced by both genetic and environmental factors. The uniqueness of the MHC alleles in each individual makes the immune response vary among the MHC haplotypes. This study represents the first attempt to determine the association between LEI0258 marker and innate immune responses as measured by APPs and leukocytes in Ross 308 broiler chickens. Increasing knowledge about the genetic nature of immune responses would provide reference data for disease‐resistant genetic improvement and molecular breeding in livestock. In the present study, we successfully identified specific alleles associated with SAA, AGP, H/L ratio, monoytosis, and basopenia.

## Author Contributions

**Afra Vatankhah**: conceptualization, investigation, validation, resources, writing–original draft preparation. **Gholamreza Nikbakht Brujeni**: conceptualization, supervision, validation, visualization, funding acquisition, methodology, formal analysis, project administration, data curation, resources, writing–review and editing. **Atefeh Esmailnejad**: formal analysis, methodology, writing–review and editing.

## Ethics Statement

The authors confirm that the ethical policies of the journal, as noted on the journal's author guidelines page, have been adhered to and the appropriate ethical review committee approval has been received (39/6/7502015).

## Conflicts of Interest

The authors declare no conflicts of interest.

### Peer Review

The peer review history for this article is available at https://publons.com/publon/10.1002/vms3.70062.

## Data Availability

The data that support the findings of this study are available from the corresponding author upon reasonable request.

## References

[vms370062-bib-0002] Bigabwa, B. A. , S. G. Nyanjom , M. Kyallo , J. Juma , J. B. D. Entfellner , and R. Pelle . 2022. “Diversity and Population Structure of Indigenous Chicken in Congo, Using MHC‐Linked Microsatellite LEI0258.” Animal Production Science 63, no. 3: 213–226.

[vms370062-bib-0003] Bonneaud, C. , M. Richard , B. Faivre , H. Westerdahl , and G. Sorci . 2005. “An MHC Class I Allele Associated to the Expression of T‐Dependent Immune Response in the House Sparrow.” Immunogenetics 57: 782–789. 10.1007/s00251-005-0046-5. 16189664

[vms370062-bib-0004] Bonneaud, C. , J. S. Sinsheimer , M. Richard , O. Chastel , and G. Sorci . 2009. “MHC Polymorphisms Fail to Explain the Heritability of Phytohaemagglutinin‐Induced Skin Swelling in a Wild Passerine.” Biology Letters 5, no. 6: 784–787. 10.1098/rsbl.2009.0435. 19671600 PMC2827992

[vms370062-bib-0005] Branton, S. L. , J. D. May , B. D. Lott , and W. R. Maslin . 1997. “Various Blood Parameters in Commercial Hens Acutely and Chronically Infected With Mycoplasma Gallisepticum and Mycoplasma Synoviae.” Avian Diseases 41: 540–547. 10.2307/1592143. 9356698

[vms370062-bib-0006] Buta, A. , S. Spătariu , O. Oprea , Z. Daradics , O. Tamas Krumpe , and L. Ognean . 2019. “Current Data Regarding the Evolution of Hematological Profile in Broiler Chickens: A Review.” Lucrări Științifice 62, no. 2: 172–179. https://repository.uaiasi.ro/xmlui/handle/20.500.12811/719.

[vms370062-bib-0007] Cerón, J. J. , P. D. Eckersall , and S. Martínez‐Subiela . 2005. “Acute Phase Proteins in Dogs and Cats: Current Knowledge and Future Perspectives.” Veterinary Clinical Pathology 34, no. 2: 85–99. 10.1111/j.1939-165X.2005.tb00019.x. 15902658

[vms370062-bib-0008] Chamanza, R. , L. van Veen , M. T. Tivapasi , and M. J. M. Toussaint . 1999. “Acute Phase Proteins in the Domestic Fowl.” World's Poultry Science Journal 55, no. 1: 67–71. 10.1079/wps19990005.

[vms370062-bib-0009] Chang, C. S. , C. F. Chen , C. Berthouly‐Salazar , et al. 2012. “A Global Analysis of Molecular Markers and Phenotypic Traits in Local Chicken Breeds in Taiwan.” Animal Genetics 43, no. 2: 172–182. 10.1111/j.1365-2052.2011.02226.x. 22404353

[vms370062-bib-0010] Clark, P. 2015. “Observed Variation in the Heterophil to Lymphocyte Ratio Values of Birds Undergoing Investigation of Health Status.” Comparative Clinical Pathology 24, no. 5: 1151–1157. 10.1007/s00580-014-2052-1.

[vms370062-bib-0011] Collisson, E. , L. Griggs , and Y. Drechsler . 2017. “Macrophages From Disease Resistant B2 Haplotype Chickens Activate T Lymphocytes More Effectively Than Macrophages From Disease Susceptible B19 Birds.” Developmental & Comparative Immunology 67: 249–256. 10.1016/J.DCI.2016.09.013. 27746172 PMC7102680

[vms370062-bib-0012] Cray, C. , J. Zaias , and N. H. Altman . 2009. “Acute Phase Response in Animals: A Review.” Comparative Medicine 59, no. 6: 517–526. 20034426 PMC2798837

[vms370062-bib-0013] Dunnington, E. A. , W. E. Briles , R. W. Briles , and P. B. Siegel . 1996. “Immunoresponsiveness in Chickens: Association of Antibody Production and the B System of the Major Histocompatibility Complex.” Poultry Science 75, no. 10: 1156–1160. 10.3382/ps.0751156. 8893288

[vms370062-bib-0014] Eckersall, P. D. 1995. “Acute Phase Proteins as Markers of Inflammatory Lesions.” Comparative Haematology International 5: 93–97. 10.1007/BF00638925.

[vms370062-bib-0015] Eckersall, P. D. , and R. Bell . 2010. “Acute Phase Proteins: Biomarkers of Infection and Inflammation in Veterinary Medicine.” Veterinary Journal 185, no. 1: 23–27. 10.1016/j.tvjl.2010.04.009. 20621712

[vms370062-bib-0016] Esmailnejad, A. , G. N. Brujeni , and M. Badavam . 2017. “LEI0258 Microsatellite Variability and Its Association With Humoral and Cell Mediated Immune Responses in Broiler Chickens.” Molecular Immunology 90: 22–26. 10.1016/j.molimm.2017.06.027. 28662410

[vms370062-bib-0018] Fulton, J. E. , H. R. Juul‐Madsen , C. M. Ashwell , et al. 2006. “Molecular Genotype Identification of the Gallus Gallus Major Histocompatibility Complex.” Immunogenetics 58, no. 5: 407–421. 10.1007/s00251-006-0119-0. 16738938

[vms370062-bib-0019] Fulton, J. E. , A. M. McCarron , A. R. Lund , et al. 2016. “A High‐Density SNP Panel Reveals Extensive Diversity, Frequent Recombination and Multiple Recombination Hotspots Within the Chicken Major Histocompatibility Complex B Region Between BG2 and CD1A1.” Genetics Selection Evolution 48, no. 1: 1–15. 10.1186/s12711-015-0181-x. PMC4705597 26743767

[vms370062-bib-0021] Gehad, A. E. , M. M. Mashaly , H. S. Siegel , E. A. Dunnington , and P. B. Siegel . 1999. “Effect of Genetic Selection and MHC Haplotypes on Lymphocyte Proliferation and Interleukin‐2 Like Activity in Chicken Lines Selected for High and Low Antibody Production Against Sheep Red Blood Cells.” Veterinary Immunology and Immunopathology 68, no. 1: 13–24. 10.1016/S0165-2427(99)00008-2. 10231948

[vms370062-bib-0022] Goto, R. M. , Y. Wang , R. L. Taylor Jr , et al. 2009. “BG1 has a Major Role in MHC‐Linked Resistance to Malignant Lymphoma in the Chicken.” Proceedings of the National Academy of Sciences 106, no. 39: 16740–16745. 10.1073/pnas.0906776106. PMC2757851 19805366

[vms370062-bib-0023] Gross, W. B. 1984. “Differential and Total Avian Blood Cell Counts by the Hemacytometer Method.” Avian Exotic Practice 1: 31–36.

[vms370062-bib-0024] Gross, W. B. , and H. S. Siegel . 1983. “Evaluation of the Heterophil/Lymphocyte Ratio as a Measure of Stress in Chickens.” Avian Diseases 27, no. 4: 972–979. 10.2307/1590198. 6360120

[vms370062-bib-0025] Gruys, E. , M. Obwolo , and M. Toussaint . 1994. “Diagnostic Significance of the Major Acute Phase Proteins in Veterinary Clinical Chemistry: A Review.” Veterinary Bulletin 64: 1009–1018.

[vms370062-bib-0026] Gruys, E. , M. J. M. Toussaint , T. A. Niewold , and S. J. Koopmans . 2005. “Acute Phase Reaction and Acute Phase Proteins.” Journal of Zhejiang University‐Science B 6, no. 11: 1045–1056. 10.1631/jzus.2005.B1045. 16252337 PMC1390650

[vms370062-bib-0027] Guangxin, E. , R. Sha , S. Zeng , C. Wang , J. Pan , and J. Han . 2014. “Genetic Variability, Evidence of Potential Recombinational Event and Selection of LEI0258 in Chicken.” Gene 537, no. 1: 126–131. 24374474 10.1016/j.gene.2013.12.040

[vms370062-bib-0028] Hassan, O. M. , E. Machuka , K. Martina , C. K. Tiambo , J. B. D. Entfellner , and R. Pelle . 2023. “The Major Histocompatibility Complex Region and Diversity of the Local Chicken Populations in Niger.” Journal of World's Poultry Science 2, no. 4: 47–54. 10.58803/jwps.v2i4.18.

[vms370062-bib-0029] Haunshi, S. , D. Devara , K. Ramasamy , R. Ullengala , and R. N. Chatterjee . 2020. “Genetic Diversity at Major Histocompatibility Complex and Its Effect on Production and Immune Traits in Indigenous Chicken Breeds of India.” Archives Animal Breeding 63, no. 1: 173–182. 10.5194/aab-63-173-2020. 32760784 PMC7397721

[vms370062-bib-0030] Izadi, F. , C. Ritland , and K. M. Cheng . 2011. “Genetic Diversity of the Major Histocompatibility Complex Region in Commercial and Noncommercial Chicken Flocks Using the LEI0258 Microsatellite Marker.” Poultry Science 90, no. 12: 2711–2717. 10.3382/ps.2011-01721. 22080008

[vms370062-bib-0031] Jaensch, S. , and P. Clark . 2004. “Haematological Characteristics of Response to Inflammation or Traumatic Injury in Two Species of Black Cockatoos: *Calyptorhynchus magnificus* and *C. funereus* .” Comparative Clinical Pathology 13: 9–13. 10.1007/s00580-004-0510-x.

[vms370062-bib-0032] Janmohammadi, A. , N. Sheikhi , H. H. Nazarpak , and G. N. Brujeni . 2020. “Effects of Vaccination on Acute‐Phase Protein Response in Broiler Chicken.” PLoS ONE 15, no. 2: 1–9. 10.1371/journal.pone.0229009. PMC7012403 32045459

[vms370062-bib-0033] Kaab, H. , M. M. Bain , and P. D. Eckersall . 2018. “Acute Phase Proteins and Stress Markers in the Immediate Response to a Combined Vaccination Against Newcastle Disease and Infectious Bronchitis Viruses in Specific Pathogen Free (SPF) Layer Chicks.” Poultry Science 97, no. 2: 463–469. 10.3382/ps/pex340. 29182756

[vms370062-bib-0034] Kaufman, J. 2000. “The Simple Chicken Major Histocompatibility Complex: Life and Death in the Face of Pathogens and Vaccines.” Philosophical Transactions of the Royal Society of London Series B: Biological Sciences 355, no. 1400: 1077–1084. 10.1098/rstb.2000.0645. 11186309 PMC1692814

[vms370062-bib-0035] Kaufman, J. 2022. “Innate Immune Genes of the Chicken MHC and Related Regions.” Immunogenetics 74, no. 1: 167–177. 10.1007/s00251-021-01229-2. 34697647 PMC8813856

[vms370062-bib-0036] Kean, R. P. , W. E. Briles , A. Cahaner , A. E. Freeman , and S. J. Lamont . 1994. “Differences in Major Histocompatibility Complex Frequencies After Multitrait, Divergent Selection for Immunocompetence.” Poultry Science 73, no. 1: 7–17. 10.3382/ps.0730007. 8165171

[vms370062-bib-0037] Kilgas, P. , R. Mänd , M. Mägi , and V. Tilgar . 2006. “Hematological Parameters in Brood‐Rearing Great Tits in Relation to Habitat, Multiple Breeding and Sex.” Comparative Biochemistry and Physiology Part A: Molecular and Integrative Physiology 144, no. 2: 224–231. 10.1016/j.cbpa.2006.02.038. 16616538

[vms370062-bib-0038] Lamont, S. J. 1998. “Impact of Genetics on Disease Resistance.” Poultry Science 77, no. 8: 1111–1118. 10.1093/ps/77.8.1111. 9706074

[vms370062-bib-0039] Latimer, K. S. , K. N. Tang , M. A. Goodwin , W. L. Steffens , and J. Brown . 1988. “Leukocyte Changes Associated With Acute Inflammation in Chickens.” Avian Diseases 32, no. 4: 760–772. 10.2307/1590996. 3202772

[vms370062-bib-0040] Lima‐Rosa, C. A. D. V. , C. W. Canal , Fallavena , L. B. D. Freitas , and F. M. Salzano . 2005. “LEI0258 Microsatellite Variability and Its Relationship to BF Haplotypes in Brazilian (Blue‐Egg Caipira) Chickens.” Genetics and Molecular Biology 28, no. 3: 386–389. 10.1590/S1415-47572005000300008.

[vms370062-bib-0042] Loudovaris, T. , M. R. Brandon , and K. J. Fahey . 1990. “The Major Histocompatibility Complex and Genetic Control of Antibody Response to Sheep Red Blood Cells in Chickens.” Avian Pathology 19, no. 1: 89–99. 10.1080/03079459008418659. 18679917

[vms370062-bib-0043] Lukasch, B. , H. Westerdahl , M. Strandh , et al. 2017. “Genes of the Major Histocompatibility Complex Highlight Interactions of the Innate and Adaptive Immune System.” PeerJ 5, no. 8: e3679. 10.7717/peerj.3679. 28875066 PMC5581531

[vms370062-bib-0044] Lwelamira, J. , G. C. Kifaro , P. S. Gwakisa , and P. L. M. Msoffe . 2008. “Association of LEI0258 Microsatellite Alleles With Antibody Response Against Newcastle Disease Virus Vaccine and Body Weight in Two Tanzania Chicken Ecotypes.” African Journal of Biotechnology 7, no. 6: 714–720. 10.4314/ajb.v7i6.58502.

[vms370062-bib-0045] Manjula, P. , M. Kim , S. Cho , D. Seo , and J. H. Lee . 2021. “High Levels of Genetic Variation in MHC‐Linked Microsatellite Markers From Native Chicken Breeds.” Genes 12, no. 2: 240. 10.3390/genes12020240. 33567601 PMC7915948

[vms370062-bib-0046] Marklová, E. 2007. “Inflammation and Genes.” Acta Medica‐Hradec Kralove 50, no. 1: 17–21. 17654831

[vms370062-bib-0048] Maxwell, M. , and G. Robertson . 1995. “The Avian Basophilic Leukocyte: A Review.” World's Poultry Science Journal 51, no. 3: 307–325. 10.1079/WPS19950021.

[vms370062-bib-0049] Miller, M. M. , and R. L. Taylor Jr . 2016. “Brief Review of the Chicken Major Histocompatibility Complex: The Genes, Their Distribution on Chromosome 16, and Their Contributions to Disease Resistance.” Poultry Science 95, no. 2: 375–392. 10.3382/ps/pev379. PMC4988538 26740135

[vms370062-bib-0050] Mitchell, M. A. , P. J. Kettlewell , and M. H. Maxwell . 1992. “Indicators of Physiological Stress in Broiler Chickens During Road Transportation.” Animal Welfare 1: 91–103. 10.1017/S0962728600014846.

[vms370062-bib-0052] Mpenda, F. N. , C. K. Tiambo , M. Kyallo , et al. 2020. “Association of LEI0258 Marker Alleles and Susceptibility to Virulent Newcastle Disease Virus Infection in Kuroiler, Sasso, and Local Tanzanian Chicken Embryos.” Journal of Pathogens 2020: 1–8. 10.1155/2020/5187578. PMC7168712 32328309

[vms370062-bib-0053] Nassar, F. S. 2021. “Genetic Diversity of the Major Histocompatibility Complex by Using LEI0258 Microsatellite Marker Associated With Productive Performance and Viral Diseases in Broiler Breeders.” Egyptian Poultry Science Journal 41, no. 2: 279–297. 10.21608/epsj.2021.182505.

[vms370062-bib-0054] Nei, M. 1973. “Analysis of Gene Diversity in Subdivided Populations.” Proceedings of the National Academy of Sciences 70, no. 12: 3321–3323. 10.1073/pnas.70.12.3321. PMC427228 4519626

[vms370062-bib-0055] Ochs, C. L. , and R. D. Dawson . 2008. “Patterns of Variation in Leucocyte Counts of Female Tree Swallows, *Tachycineta bicolor*: Repeatability Over Time and Relationships With Condition and Costs of Reproduction.” Comparative Biochemistry and Physiology Part A: Molecular and Integrative Physiology 150, no. 3: 326–331. 10.1016/j.cbpa.2008.04.003. 18485771

[vms370062-bib-0056] Ohara, A. , C. Oyakawa , Y. Yoshihara , S. Ninomiya , and S. Sato . 2015. “Effect of Environmental Enrichment on the Behavior and Welfare of Japanese Broilers at a Commercial Farm.” Journal of Poultry Science 52, no. 4: 323–330. 10.2141/jpsa.0150034.

[vms370062-bib-0057] Oladejo, O. A. , S. O. Oseni , M. Kyallo , et al. 2023. “Evaluation of Genetic Variability in Four Nigerian Locally‐Adapted Chicken Populations Using Major Histocompatibility Complex‐Linked LEI0258 Microsatellite Marker.” Poultry Science Journal 11, no. 2: 189–201.

[vms370062-bib-0058] O'Reilly, E. L. , and P. D. Eckersall . 2014. “Acute Phase Proteins: A Review of Their Function, Behaviour and Measurement in Chickens.” World's Poultry Science Journal 70, no. 1: 27–44. 10.1017/S0043933914000038.

[vms370062-bib-0059] Owen, J. P. , M. E. Delany , C. J. Cardona , A. A. Bickford , and B. A. Mullens . 2009. “Host Inflammatory Response Governs Fitness in an Avian Ectoparasite, the Northern Fowl Mite (*Ornithonyssus sylviarum*).” International Journal for Parasitology 39, no. 7: 789–799. 10.1016/j.ijpara.2008.12.008. 19367920

[vms370062-bib-0061] Saeki, K. , and T. Yokomizo . 2017. “Identification, Signaling, and Functions of LTB4 Receptors.” Seminars in Immunology 33: 30–36. 10.1016/j.smim.2017.07.010. 29042026

[vms370062-bib-0062] Schat, K. A. , R. L. Taylor Jr , and W. E. Briles . 1994. “Resistance to Marek's Disease in Chickens With Recombinant Haplotypes of the Major Histocompatibility (B) Complex.” Poultry Science 73, no. 4: 502–508. 10.3382/ps.0730502. 8202429

[vms370062-bib-0063] Schou, T. W. , R. Labouriau , A. Permin , et al. 2010. “MHC Haplotype and Susceptibility to Experimental Infections (*Salmonella* Enteritidis, *Pasteurella multocida* or *Ascaridia galli*) in a Commercial and an Indigenous Chicken Breed.” Veterinary Immunology and Immunopathology 135, no. 1–2: 52–63. 10.1016/J.VETIMM.2009.10.030. 19945754

[vms370062-bib-0064] Shini, S. , P. Kaiser , A. Shini , and W. L. Bryden . 2008. “Differential Alterations in Ultrastructural Morphology of Chicken Heterophils and Lymphocytes Induced by Corticosterone and Lipopolysaccharide.” Veterinary Immunology and Immunopathology 122, no. 1–2: 83–93. 10.1016/j.vetimm.2007.10.009. 18045696

[vms370062-bib-0065] Silva, A. P. D. , and R. A. Gallardo . 2020. “The Chicken MHC: Insights Into Genetic Resistance, Immunity, and Inflammation Following Infectious Bronchitis Virus Infections.” Vaccines 8, no. 4: 637. 10.3390/vaccines8040637. 33147703 PMC7711580

[vms370062-bib-0066] Smith, J. , J. R. Sadeyen , D. Cavanagh , P. Kaiser , and D. W. Burt . 2015. “The Early Immune Response to Infection of Chickens With Infectious Bronchitis Virus (IBV) in Susceptible and Resistant Birds.” BMC Veterinary Research 11: 1–14. 10.1186/S12917-015-0575-6. 26452558 PMC4600211

[vms370062-bib-0068] Tizard, I. R. 2013. “Systemic Responses to Inflammation.” In Veterinary Immunology, 52–60. Saunders, Philadelphia: Elsevier Health Sciences.

[vms370062-bib-0069] Xu, W. , and A. Larbi . 2018. “Immunity and Inflammation: From Jekyll to Hyde.” Experimental Gerontology 107: 98–101. 10.1016/j.exger.2017.11.018. 29187316

[vms370062-bib-0070] Yeh, F. , R. Yang , T. Boyle , and J. Mao . 1997. “POPGENE Software: Microsoft Windows‐Based Freeware for Population Genetic Analysis (version 1.32).” In Center for International Forestry Research. Edmonton, Canada: University of Alberta.

[vms370062-bib-0071] Zekarias, B. , A. A. Ter Huurne , W. Landman , J. Rebel , J. Pol , and E. Gruys . 2002. “Immunological Basis of Differences in Disease Resistance in the Chicken.” Veterinary Research 33, no. 2: 109–125. 10.1051/vetres:2002001. 11944802

[vms370062-bib-0072] Zhou, H. , and S. J. Lamont . 2003. “Chicken MHC Class I and II Gene Effects on Antibody Response Kinetics in Adult Chickens.” Immunogenetics 55: 133–140. 10.1007/s00251-003-0566-9. 12743657

